# Optical Coherence Tomography Predictors of Favorable Functional Response in Naïve Diabetic Macular Edema Eyes Treated with Dexamethasone Implants as a First-Line Agent

**DOI:** 10.1155/2021/6639418

**Published:** 2021-03-24

**Authors:** Alessandro Meduri, Giovanni William Oliverio, Luigi Trombetta, Marta Giordano, Leandro Inferrera, Costantino John Trombetta

**Affiliations:** Department of Biomedical Sciences, Ophthalmology Clinic, University of Messina, Messina, Italy

## Abstract

**Purpose:**

To evaluate efficacy and safety of intravitreal dexamethasone 0.7 mg implant in treatment-naïve DME patients and to assess the utility of OCT structural biomarkers as predictors of functional response after treatment.

**Methods:**

Thirty-nine eyes of 39 diabetic patients with center involving DME were enrolled. Best-corrected visual acuity (BCVA) and SS-OCT (DRI SS-OCT Triton, Topcon, Japan) to evaluate central retinal thickness (CRT), serous retinal detachment (SRD), intraretinal cysts (IRC), number of hyper-reflective spots (HRS), integrity of the ellipsoid zone (EZ), disorganization of the inner retinal layers (DRIL), vitreomacular adhesion (VMA), vitreomacular traction (VMT), and posterior vitreous detachment (PVD) were evaluated at baseline and at 3, 6, and 12 months after treatment. Multiple logistic analysis was performed to evaluate the possible OCT biomarker as predictive factors for final visual acuity improvement at the end of treatment.

**Results:**

At 12 months after treatment, the mean BCVA improved from 51.6 ± 17.5 to 56.9 ± 17.3 ETDRS letters (*p*=0.03). Furthermore, there were statistically significant changes in CRT, IRC, HRS, and SRD. Nineteen patients presented a  >10-letters improvement in BCVA; the presence of SRD at baseline was a predictor of good functional treatment response at 12 months (OR 2.1; 95% C.I. 1.2–4.9; *p*=0.001) as well as the presence of EZ integrity preoperatively (OR 1.3; 95% C.I. 0.5–2.4; *p*=0.001) and the absence of vitreoretinal interface alteration (OR 1.1; 95% C.I. 0.3–2.3; *p*=0.02). No significant changes in the IOP and lens status were observed throughout the follow-up period.

**Conclusion:**

This study empathized the importance of structural biomarkers as predictors of favorable response and confirmed the efficacy and safety of intravitreal dexamethasone implant in treatment-naïve DME patients showing a better functional response in the presence of SRD integrity of EZ and absence of vitreoretinal alterations.

## 1. Introduction

Diabetic macular edema (DME) represents one of the major causes of visual impairment in diabetic patients due to the abnormal collection of intra- and/or subretinal fluid in the macular area caused by the alteration of the blood-retinal barrier [1]. DME is classified according to its etiology as vasogenic or nonvasogenic as recent studies highlighted the central role of inflammation in the pathogenic mechanisms [2–4].

The advances in optical coherence tomography (OCT) technology have contributed to improving our understanding of the pathophysiology and classification of diabetic macular edema and have enabled us to recognize structural biomarkers for a morphologic categorization of the disease that can influence treatment outcome [2, 5–9].

DME presents with different patterns on OCT including sponge-like swelling cystoid macular edema and serous retinal detachment (SRD) [10]. Additionally, subfoveal thickness (CST), the presence of hyper-reflective spots (HRS), the presence of SRD the size of intraretinal cysts (IRC), the occurrence of disorganization of the inner retinal layers (DRIL), the state of the ellipsoid zone (EZ), the external limiting membrane (ELM), and choroidal thickness (CT) have been used to categorize and grade DME [8]. Furthermore, the effectiveness of dexamethasone intravitreal implant 0.7 mg in the treatment of DME has been demonstrated in several studies; however, there is a lack in literature regarding the prognostic factors after treatment in particular in treatment-naïve DME patients [11, 12].

The aim of this study is to evaluate the efficacy and safety of intravitreal dexamethasone implant in treatment-naïve DME patients and to assess the utility of OCT structural biomarkers as predictors of functional response after treatment.

## 2. Methods

In this study, data from 39 eyes of 39 diabetic patients with DME were retrospectively analyzed. Patients aged >18 years and treatment-naïve DME with a central macular thickness (CMT) ≥ 300 *µ*m who received an intravitreal implant of dexamethasone 0.7 mg (Ozurdex® Allergan. Inc. Irvine California USA) were enrolled.

Patients with a history of vitreoretinal surgery cataracts other macular diseases glaucoma and iris rubeosis were excluded.

Informed consent was obtained from all patients after the explanation of nature and the possible consequences of the study. This study was approved by the Institutional Review Board of the University of Messina, and it was conducted in accordance with the tenets of the Declaration of Helsinki.

Anamnestic data were reported for each patient including type and duration of diabetes and value of the recent glycated hemoglobin (HbA1c). A complete ophthalmologic assessment was carried out comprising best-corrected visual acuity (BCVA) using the Early Treatment Diabetic Retinopathy Study (ETDRS) chart microscopic evaluation of the anterior segment applanation tonometry and swept-source OCT (DRI SS-OCT Triton, Topcon, Japan).

These data were taken at each visit prior to the intravitreal dexamethasone implant and after 3, 6, and 12 months from the treatment.

### 2.1. Optical Coherence Tomography Analysis

SS-OCT images were obtained using a 9-mm radial OCT scan centered on the fovea. Automatic analysis using the OCT software IMAGEnet 6 (version 1.17.9720; Topcon Medical Systems Inc., Oakland, NJ, USA) was performed to evaluate the structural retinal biomarkers such as the presence of SRD, intraretinal cysts (IRC), continuity of the ellipsoid zone (DRIL), vitreomacular adhesion (VMA) or traction (VMT), and posterior vitreous detachment (PVD).

**Figure 1:**
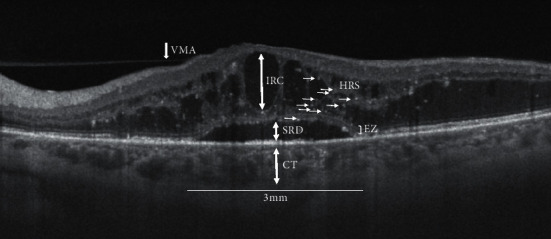
Swept-source optical coherence tomography radial scan (9 mm) of a patient with diabetic macular edema and subfoveal neuroretinal detachment showing structural biomarkers evaluated within a 3 mm area centered on the fovea: CT: choroidal thickness; SRD: serous retinal detachment; EZ: ellipsoid zone integrity; IRC: intraretinal cysts; HRS: hyper-reflective spots; VMA: vitreomacula adhesion.

A count of the total HRS was performed and calculated in the area of 3 mm centered on the fovea (Figure 1).

The height of serous retinal detachment (SRD) was manually calculated using the built-in caliper tool of the instrument as the space between the outer retinal and the RPE surfaces at the fovea (Figure 1).

The height of IRC was measured summing all individual cyst heights within 3 mm of the fovea to give a total height value (Figure 1).

Choroidal thickness (CT) was manually analyzed on the foveal center and on temporal and nasal site tracing two vertical lines at 1.5 mm temporally from the fovea and 1.5 mm nasally from the fovea (Figure 1).

### 2.2. Statistical Analysis

The fitting of the data to a normal distribution was tested by the Kolmogorov–Smirnov test. In order to evaluate the existence of statistically significant differences in different times of observation, we applied the Wilcoxon test signed-rank test and Student's *t*-test for paired data as appropriate (for numerical variables) and the McNemar test (for dichotomous data). Logistic regression analysis was performed to evaluate the possible OCT biomarker (CRT, IRC, CT, SRD, HRS, EZ, and vitreomacular alterations) as predictive factors for final visual acuity improvement at the end of treatment.

A *p* value smaller than 0.05 was considered to be statistically significant. Statistical analyses were performed using the SPSS 26.0 for the macOS package.

## 3. Results

### 3.1. Study Population

Thirty-nine patients (19 males 20 females) with DME were enrolled in this study. The mean age of patients was 66.7 ± 7.3 years, the mean duration of diabetes was 13.5 ± 7.3 years, and the mean recent Hba1c was 7.9 ± 3.2% (Table 1). Twenty-one patients (53.8%) presented a cystoid macular edema, and 18 patients (46.2%) presented a subfoveal neuroretinal detachment.

### 3.2. Functional and Morphological Outcome

Baseline and posttreatment data are reported in Table 2. At 3 months, the mean BCVA improved from 51.6 ± 17.1 to 58.9 ± 16.5 ETDRS letters (*p*=0.01) and to 57.6 ± 17.3 and to 56.9 ± 17.5 at 6 and 12 months, respectively (*p*=0.03). Furthermore, there were statistically significant changes in CRT, IRC dimension, HRS number, and SRD height after treatment throughout the follow-up (Table 2). No significant changes in CT were observed after treatment. At the end of follow-up, 15 patients presented a complete resolution of the SRD.

Nineteen patients (48.7%) presented a >10-letters improvement in BCVA at the end of follow-up.

At 6 months, a second dexamethasone implant was necessary in 5 patients (12.8%).

No significant increments of the mean IOP were observed. Overall, the follow-up period and no changes in the lens status were recognized after treatment.

### 3.3. Optical Coherence Tomography Predictors for Treatment Response

The presence of SRD at baseline was a predictor of good functional treatment response at 12 months (OR 2.1; 95% C. I. 1.2–4.9; *p* < 0.001). Additionally, the presence of EZ integrity preoperatively was a predictor of good functional treatment response at 12 months (OR 1.3; 95% C. I. 0.5–2.4; *p*=0.001). Eyes without vitreoretinal interface alteration at baseline presented a better functional outcome at 12 months after treatment (OR 1.1; 95% C. I. 0.3–2.3; *p*=0.02). There was no significant correlation between DRIL presence number of HRS IRC dimension CT and a >10-letters improvement in BCVA.

## 4. Discussion

Recent studies have demonstrated the primary role of inflammatory and vascular factors in the pathogenesis and development of DME; however, these mechanisms are complex and still not completely clarified [2–4]. The neurovascular unit consists of Müller cells, astrocytes, ganglion cells, and amacrine cells in a dynamic interaction with retinal vascular endothelial cells and pericyte-releasing factors that induce the formation of tight junctions in retinal vessels [13–15]. Abnormalities in Müller cells probably affect this barrier property in the retinal vessels in diabetic patients [13, 14]. Indeed, blood-retinal barrier disruption is associated with an increase of vascular endothelial growth factor (VEGF), intercellular adhesion molecule-1 (ICAM-1), interleukin-6 (IL-6), and monocyte chemotactic protein-1 (MCP-1) among others [14, 16]. Furthermore, in the recent years, numerous advances have been made in the treatment of diabetic retinopathy and DME [14–17]. Anti-VEGF is considered first-line treatment; however, corticosteroids represent a fundamental alternative for treating these patients [10, 18]. The efficacy of corticosteroids in DME may be attributable to the strong anti-inflammatory and antiedema properties of these molecules as previous studies demonstrated the reduced expression of VEGF and other inflammatory mediator-diminished leukostasis and vascular leakage finally improving the barrier function of endothelial cell tight junction [19]. Corticosteroids are mainly used as a second choice due to the possible adverse events occurrence such as increase of IOP and cataract progression [10, 18]. Additionally, corticosteroids are useful for the treatment of refractory forms of DME to anti-VEGF [10, 18].

Nevertheless, the intravitreal implant of dexamethasone could represent a first-line therapy in particular conditions such as patients with a recent history of major cardiovascular events and contraindications to anti-VEGF therapy patients with vitrectomized eye, pregnancy, pseudophakic patients, and uncompliant patients, unable or unwilling to return for regular examinations [10, 18].

Although numerous studies confirmed the efficacy and safety of intravitreal implant of dexamethasone, there is a lack in literature about the outcome and the predictive factors in treatment-naïve patients with DME [18]. The introduction of OCT improved the structural evaluation of the retinal layers introducing several morphological biomarkers that could help to assess and predict the functional outcome and to choose the best treatment for the patient [5, 10].

Several studies demonstrated that macular thickness may serve as a measurement variable in relationship with treatment outcome in DME [5, 10]. Furthermore, recent studies have demonstrated that the presence of SRD and HRS are correlated with high inflammatory component [5–7].

Vujosevic et al. showed that DME patients with SRD and a high number of HRS presented a better response to intravitreal dexamethasone rather than anti-VEGF [6].

In this study, we have evaluated the long-term result of intravitreal implant of dexamethasone in a group of treatment-naïve DME patients. According to previous studies, we have demonstrated the effectiveness of this treatment as a first-line showing a long-term morphological and functional improvement in DME patients. Zur et al. identified the presence of SRD, EZ continuity, absent HRS, and an attached vitreoretinal interface as biomarkers that predict a better visual acuity improvement after dexamethasone implants in eyes with DME [5].

In our study, at 6 and 12 months, the BCVA and the CMT improved significantly after treatment. Additionally, in the subgroup analysis, patients with preoperative SRD presented a better functional improvement at 6 and 12 months, and the presence of the EZ integrity was associated with a better visual outcome at the end of the follow-up.

However, there was no correlation between DRIL presence, number of HRS, IRC, CT, and visual acuity improvement at 6 and 12 months. Rosenblatt et al. in a multicentric study reported an improvement of >10-letters in BCVA in 46.1% in a treatment-naïve group; this result was in accordance with our findings [12].

The main limitations of this study are the small sample size and the retrospective design.

In conclusion, this study confirmed the efficacy and safety of intravitreal dexamethasone implant in the treatment of naïve DME patients and demonstrated a better functional response in patients with the presence of SRD and EZ integrity and absence of vitreomacular alterations; however, further studies are necessary to assess the usefulness of OCT structural biomarker as predictors of functional response in DME patients. Additionally, our findings emphasized the importance of an accurate evaluation of structural biomarkers to choose the best treatment for the patient.

## Figures and Tables

**Table 1:** Clinical characteristics of the study population.

Variables	
Age (years)	65.2 ± 11.3
Gender (male/female)	19/20
Duration of diabetes (years)	13.5 ± 7.3
HbA1c (%)	7.9 ± 3.2
Lens status Phakic (*n*) Psuedophakic (*n*)	12 27

HbA1c: glycated hemoglobin.

**Table 2:** Functional and morphological biomarkers before and after treatment.

Variables	Baseline	3 months	*p* value Baseline vs. 3 Mo	6 months	*p* value Baseline vs. 6 Mo	12 months	*p* value Baseline vs. 12 Mo
BCVA ETDRS letters	51.6 ± 17.1	58.9 ± 16.5	**0.01**	57.6 ± 17.3	**0.03**	56.9 ± 17.5	**0.03**
CMT (*µ*m)	434.4 ± 155.6	284.7 ± 108.7	**<0.001**	284.7 ± 108.7	**0.001**	336.8 ± 151.1	**0.001**
SRD presence, *n* (%) Height (µm)	18 (46.2) 32.6 ± 52.7	2 (5.1) 6.5 ± 22.8	**<0.001** **<0.001**	3 (7.7) 13.5 ± 32.1	**<0.001** **0.001**	3 (7.7) 14.1 ± 28.1	**<0.001** **0.001**
HRS (*n*)	25.5 ± 13.8	16.2 ± 15.2	**0.02**	18.7 ± 12.6	**0.03**	25.7 ± 12.6	**0.03**
Cysts size (*µ*m)	2216.1 ± 1230.9	631.1 ± 763.1	**0.001**	1384.4 ± 952.9	**0.001**	1384.4 ± 952.9	**0.01**
CT Subfoveal (*µ*m) Nasal (*µ*m) Temporal (*µ*m)	178.6 ± 65.9 155.8 ± 31.8 163.4 ± 39.7	178.6 ± 65.9 155.8 ± 31.8 163.4 ± 39.7	0.32 0.44 0.32	178.6 ± 65.9 155.8 ± 31.8 163.4 ± 39.7	0.41 0.15 0.31	178.6 ± 65.9 155.8 ± 31.8 163.4 ± 39.7	0.19 0.28 0.33
DRIL presence, *n* (%)	8 (20.5)	7 (17.9)	0.77	7 (17.9)	0.77	7 (17.9)	0.77
EZ integrity, *n* (%)	23 (58.9)	22 (56.4)	0.82	22 (56.4)	0.82	22 (56.4)	0.82
Vitreomacular interface PVD, *n* (%) VMA, *n* (%) VMT, *n* (%)	7 (17.9) 11 (28.2) 4 (10.3)	7 (17.9) 11 (28.2) 3 (7.7)	— — 0.69	8 (20.5) 11 (28.2) 3 (7.7)	0.77 — .69	8 (20.5) 11 (28.2) 3 (7.7)	0.77 — .69

BCVA: best-corrected visual acuity; ETDRS: Early Treatment Diabetic Retinopathy study; CMT: central macular thickness; SRD: serous retinal detachment; HRS: hyper-reflective spots; CT: choroidal thickness; DRIL: disorganization of the inner retinal layers; EZ: ellipsoid zone; PVD: posterior vitreous detachment; VMA: vitreomacular adhesion; VMT: vitreomacular traction. Values in bold indicate *p* value < .05.

## Data Availability

The data used to support the findings of this study are available from the corresponding author upon request.
